# An Explorer of Chemical Biology of Plant Natural Products in Southwest China, Xiaojiang Hao

**DOI:** 10.1007/s13659-018-0184-8

**Published:** 2018-07-21

**Authors:** Yue-mao Shen, Duo-zhi Chen

**Affiliations:** 1 School of Pharmaceutical Sciences, Shandong University, Jinan, 250012 People’s Republic of China; 2 State Key Laboratory of Functions and Applications of Medicinal Plants, Academic City, No. 3491 Platina Way, Hi-tech Zone, Guiyang, Guizhou 550014 People’s Republic of China; 3 State Key Laboratory of Phytochemistry and Plant Resources in West China, Kunming Institute of Botany, Chinese Academy of Sciences, Kunming, 650201 People’s Republic of China

**Keywords:** Natural products science, Medicinal chemistry, Chemical biology, Chemical ecology

## Abstract

**Abstract:**

Xiaojiang Hao, who obtained Master Degree from Kunming Institute of Botany (KIB), Chinese Academy of Sciences (CAS) in 1985, and Doctor in Pharmacy degree in Pharmacy from Institute for Chemical Research, Kyoto University, in 1990, was born in Chongqing in July, 1951. In 1991, he returned to KIB, CAS, as an Associate professor and served as the chair of the Department of Phytochemistry. In 1994, he was promoted to a full professor at the current institute. He served as the Deputy Director of KIB and the Director of Open Laboratory of Phytochemistry from 1995 to 1997, and the Director of KIB from 1997 to 2005. Professor Hao has published more than 450 peer-reviewed SCI papers, which have been cited over 6000 times. He has obtained one PCT patent and 23 patents in China. Due to his tremendous efforts, one candidate drug, phenchlobenpyrrone, has entered the Phase II clinical trail for the treatment of Alzheimer’s disease. Moreover, he won the First Prize of Natural Sciences in Yunnan Province for three times, and Ho Leung Ho Lee Fund Science and Technology Innovation Award in 2017.

**Graphical Abstract:**

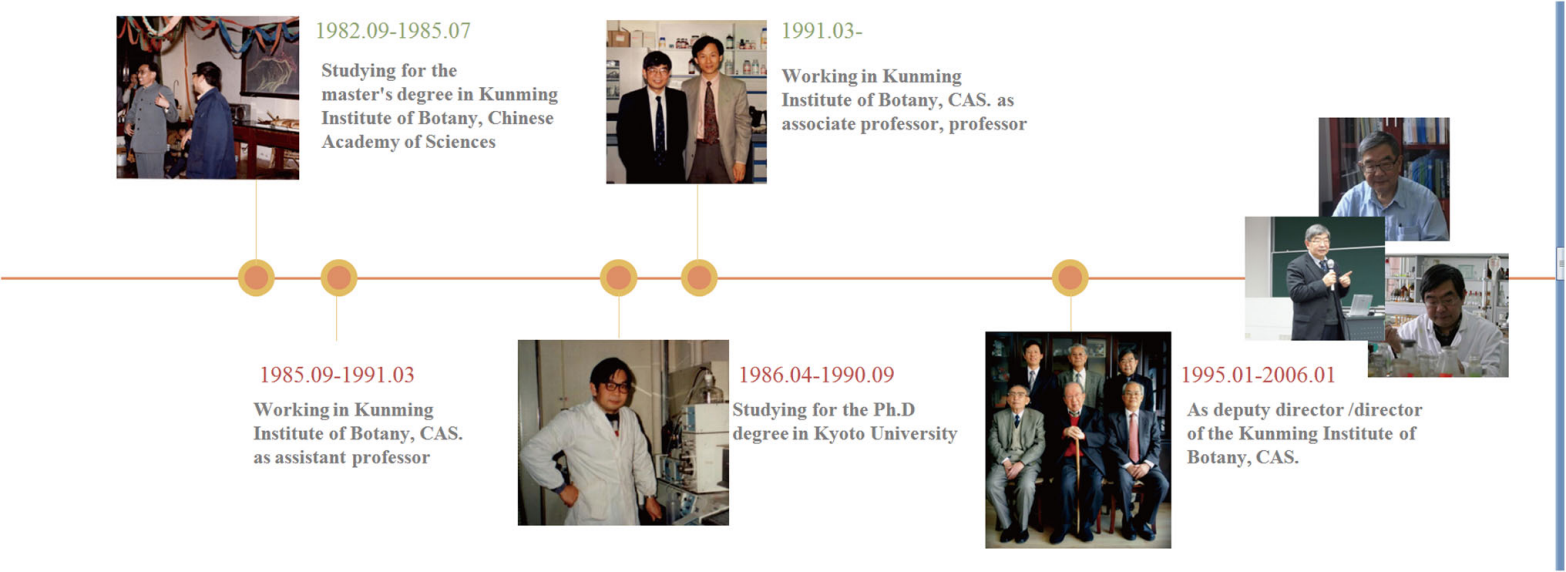

## Introduction

China is one of the countries in the world with the most abundant plant resources, and Yunnan Province is known as the “Kingdom of Plants” as well as the “Treasure house of Drugs”. A wide variety of medicinal plants are the cornerstones of the formation and development of Chinese medicine. China has long lived in harmony with nature. The rich natural resources and diverse plants have brought endless supply for medicinal herbs to the Chinese people. With the development of modern medical sciences, phytochemistry is also playing an increasingly important role. For 40 years, Professor Hao has been an enthusiastic explorer in the field of Natural Product Sciences of Plants and written a profound chapter for the plant resources in Southwest China.

Professor Hao has dedicated his life to medicinal plant chemistry and natural product research to meet the health care needs of the country. After graduating from Department of Chemistry at Guizhou University in 1976, he began to engage in phytochemical research and quickly realized the great potential and prospects of medicinal plant chemistry. He determined to continue his studies in this area from the very beginning of his career. He was admitted to the graduate program at KIB in 1982, and after receiving his MS degree in science, he stayed at KIB as an Assistant Professor. In 1986, he was selected to study at Institute of Chemical Research, Kyoto University in Japan. Four years later, he received PhD in Pharmacy with outstanding achievements and immediately returned to the motherland, where he applied his knowledge and expertise to the needs of the motherland.

After returning to China, Professor Hao took the responsibility for the construction of disciplines in phytochemistry. He was appointed as an Associate Professor and the chair of Department of Phytochemistry of KIB from March 1991 to April 1994 and became a Professor. Anybody can be a Researcher and supervisor for master students and doctoral students since May 1994. From January, 1995 to November, 1997, he was the executive deputy director of KIB and the director of the Open Laboratory of Plant Chemistry. And from the year of 1997 to 2005, he served as the director of KIB. Under his leadership, KIB applied for “Research and Development Base for Biological Resources and Biodiversity Conservation in Southwest China” in 1998, which was a pilot program for the Knowledge Innovation Project of the Chinese Academy of Sciences. Then in November 2001, he led the successfully application of the State Key Laboratory of Plant Chemistry and Western Plant Resources, where he served as the first director of the State Key Laboratory. In 1998, entrusted by the Guizhou Provincial Government and CAS, Professor Hao took the responsibility to establish the “Key Laboratory of Natural Product Chemistry of the Guizhou Province-CAS”, which was approved in 2003 as the first batch of breeding bases for state key laboratories jointly established by Provinces and the Ministry of Science and Technology. In 2016, the Ministry of Science and Technology approved the establishment of the State Key Laboratory for the Efficacy and Utilization of Medicinal Plants. In spite of the heavy daily administrative work, Professor Hao always insists on the scientific research and graduate student training. His research team has always been at the front-line of his research field. His hardwork and efforts have produced a series of important research achievements and cultivated a new generation of natural product researchers. He has published over 450 papers in SCI-original academic journals which has been cited more than 6000 times. Additionally, he is granted with 1 PCT international patent which was licensed to a company and 23 national invention patents.

## Mining Novel Phytochemical Structures from Diverse Plants in Southwest China

**Fig. 1:**
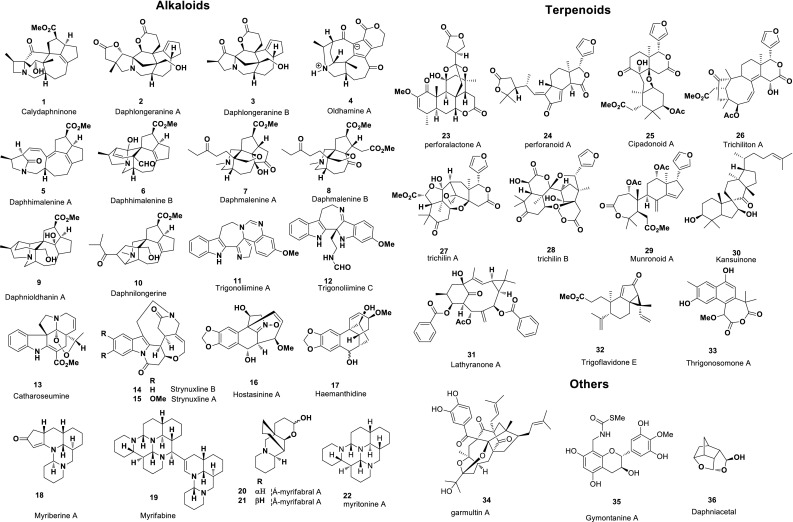
Thirty six natural products from the Hao’s group were cited by “Hot off the Press” of *Natural Product Reports* [1–32]

Since 1991, the Hao’s team has studied the chemical components of more than 200 species of plants and identified more than 4600 chemical constituents including 1400 new structures and 90 new scaffolds, part of which were cited by “Hot off the Press” in *Natural Product Reports* (Fig. 1) [1–32]. The studies include the elucidation of complex chemical structures, exotic and novel backbones, and novel biogenetic mechanisms for *Daphniphyllum* alkaloids. The Hao Lab has become one of the most important discovers of new *Daphniphyllum* alkaloids [24–62]. “The research on novel structure alkaloids of *Daphniphyllum*” he presided over won the First Class Award of Natural Science of Yunnan Province in 2009. These results were actually inspired by the hypothesis of the biosynthetic pathway of *Daphniphyllum* alkaloids that is the “premature” involvement of amines in the formation of molecular skeletons, which lead to the structural diversity of *Daphniphyllum* alkaloids in *Daphniphyllum* species. The structural diversity of plant natural products derived from the diversity of biosynthetic pathways is also mirrored by other new natural products, such as alkaloids [63–80], terpenoids [81–113], polyphenols [114–123], and steroids [124–128] rooted from the plentiful plant resources in Southwest China.

## Exploring Phytochemical Geography of the *Spiraea japonica* Complex

**Fig. 2:**
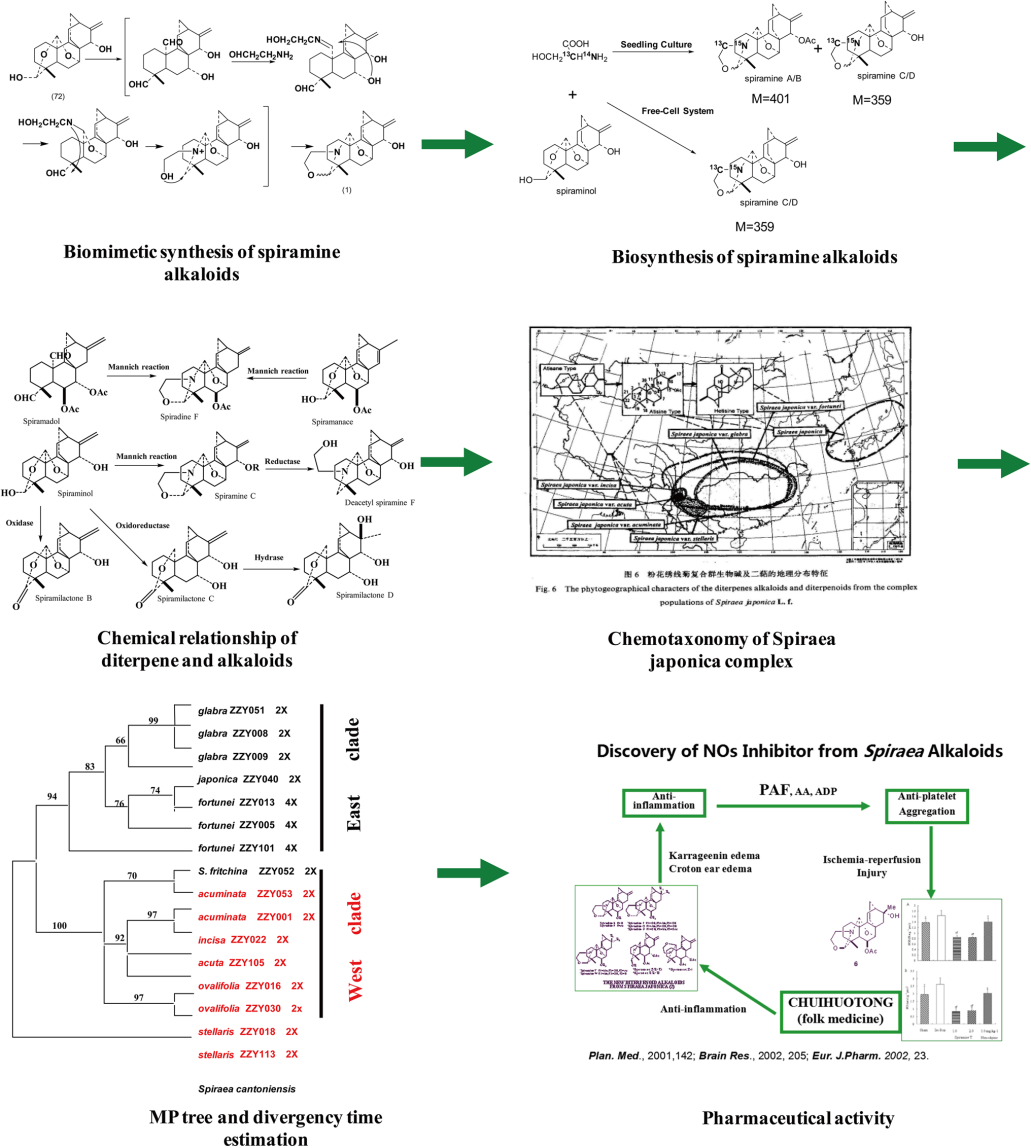
Chemical and biological studies reveal a phytochemical geographic feature of the *Spiraea japonica* complex

In his long history of scientific research practice, Professor Hao never hesitated to learn new knowledge and explore beyond his comfortable zones, which has led him to new research systems and strategies and helped him continuously achieve innovative results. For example, he discovered that only the compound of *Spiraea japonica* L.f. contained diterpenes and diterpene alkaloids among more than 100 species of the genus *Spiraea*. This unusual phenomenon prompted him to select the *Spiraea japonica* compound to carry out exploratory research by combining plant resources and distribution, diterpenoids and alkaloids as well as biology to reveal several scientific issues of plant populations at different levels. He discovered and carried out the biomimetic and biosynthetic pathways from diterpenoids to diterpene alkaloids. These results revealed a high degree of harmonization and correlations between plant population evolution, plant faunal evolution, and chemical composition types, which exemplified a novel research model of phytochemistry (Fig. 2) [128–167]. This research won the First Class Award of Natural Science of Yunnan Province in 2003.

## Harnessing the Potential of Plant Chemical Defenses from Antiviral Activities

**Fig. 3:**
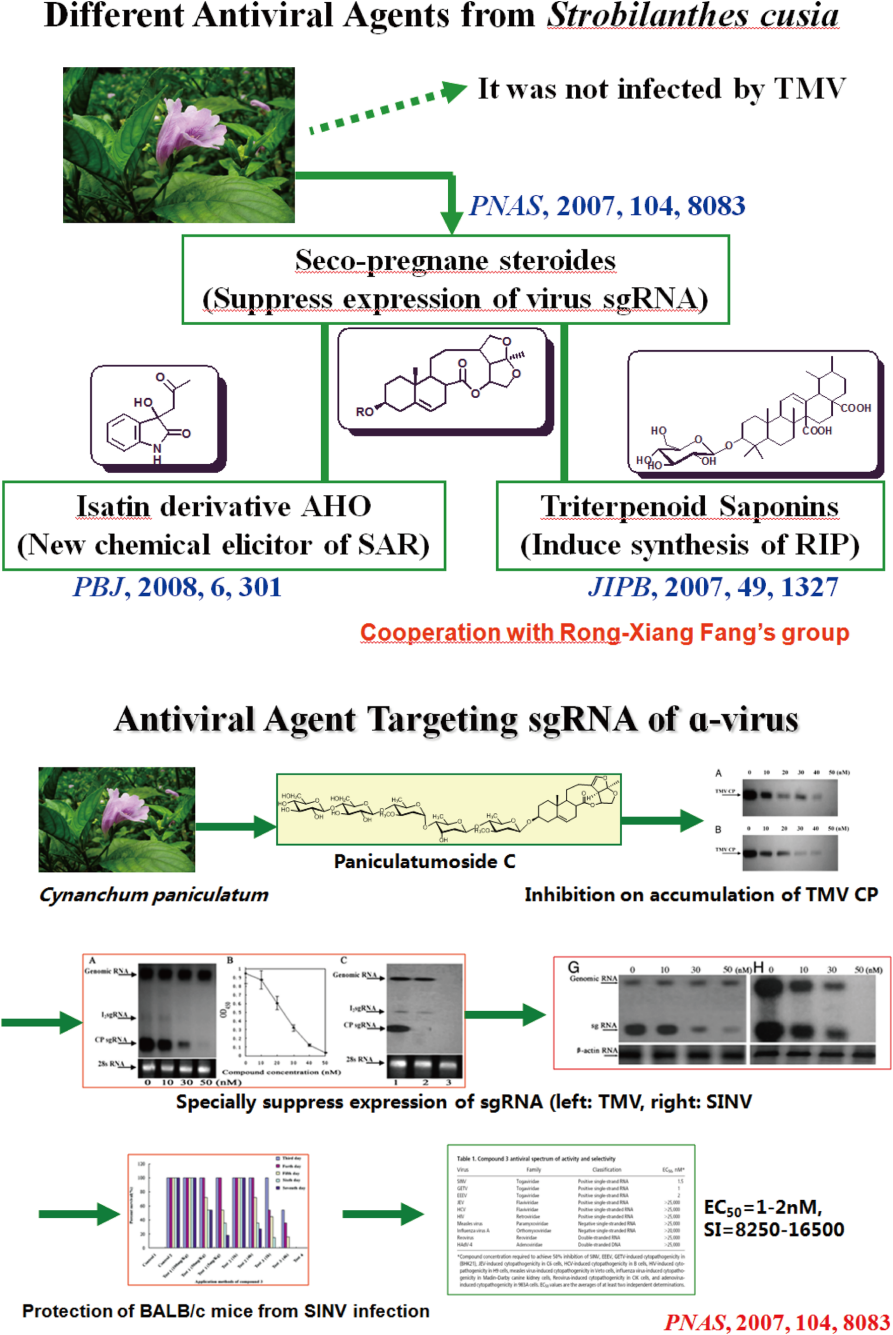
Studies on low-toxic plant chemical defense molecules

For a long time, plant secondary metabolites (natural products) have been considered to have no biological significance. Since 2002, Professor Hao has proposed that “Low-toxic plant metabolites may have a role in chemical defense of plants against viruses” and subsequently found three types of plant viral antagonists from *Strobilanthes cusia* and *Cynanchum paniculatum* [168–186]. Since then, over 20 types of plant-derived pesticide lead compounds with potential for development of plant resistance against viruses and bacterial pathogens have been discovered in plants. The new research directions in plant chemical defense have been established in the Hao Lab (Fig. 3) [50–67], and the research has led to the First Class Award of Natural Science of Yunnan Province in 2013.

## Approaching Chemical Biology with Natural Chemical Probes

**Fig. 4:**
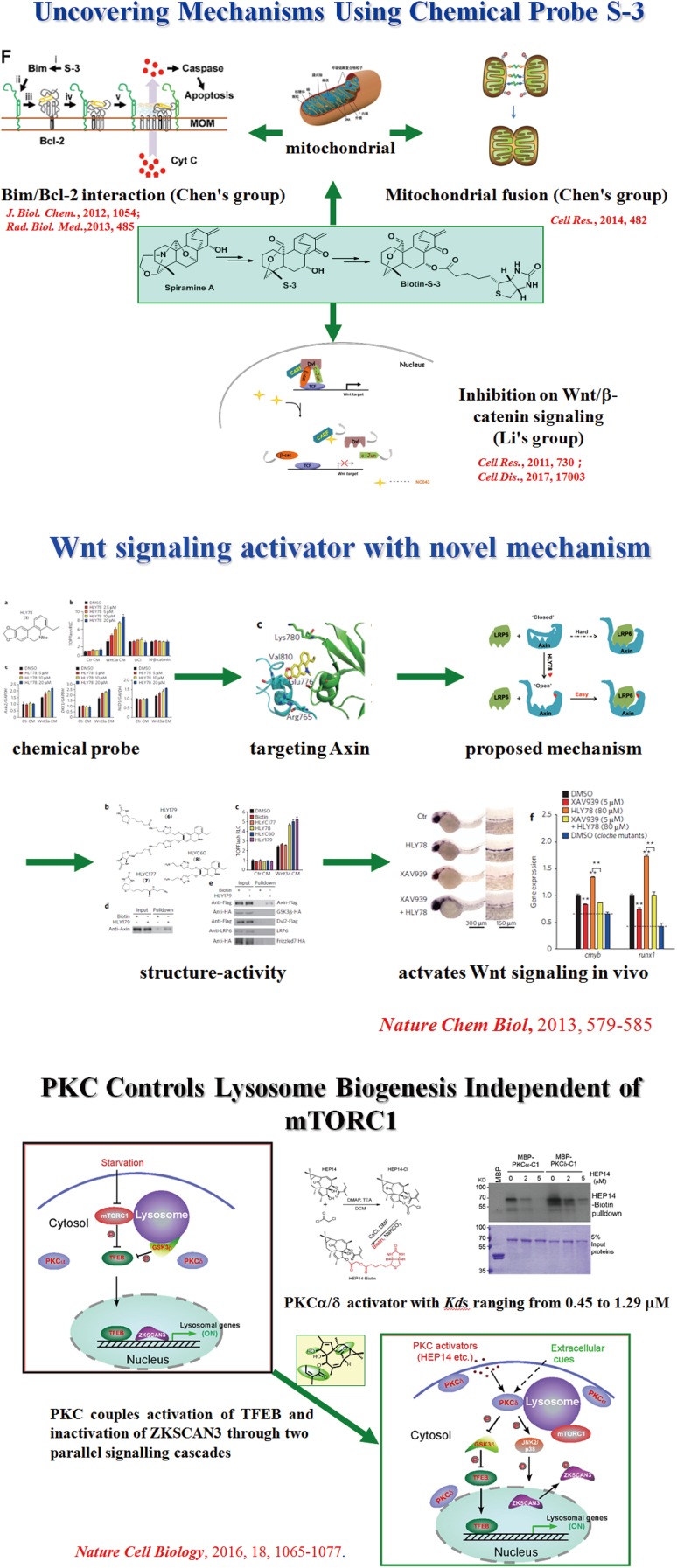
Cooperative research of Prof. Hao on chemical biology of plant natural products

Although Professor Hao’s training background is natural products chemistry, most books in his office books are biological. Whenever he has the chance, he would seize it to absorb new knowledge in biology. If he did not understand, he would consult a biologist or graduate student engaged in biological research. He often said that the rapid development of the life sciences in the 21st century would surely bring new opportunities to the development of the ancient discipline of natural products chemistry. For more than a decade, he has actively promoted co-operations with biologists, developed the research direction of chemical biology with the use of small molecule natural products as chemical probes, and obtained preliminary results in the research of the mechanism of action related to tumors, neuroprotection, and viruses [187–201]. New areas of chemical biology research have been established with biologists including Prof. Li Lin [187, 190, 192, 196, 200], Prof. Chen Quan [194, 195, 197, 199, 201] and Prof. Yang Chonglin [188, 191]. The collaborations have provided new strategies, new potential targets, and lead compounds for the treatment of related diseases (Fig. 4).

Although the research interests are in many areas, the main thrust of Professor Hao’s efforts has always been the study and development of innovative drugs and pesticides. Hao designed and synthesized a new anti-AD drug called Fenkeluotong with independent intellectual property rights. Phase II clinical trail is underway for this candidate drug. He directed the research on the mechanism of action, large-scale synthetic technology, safety evaluation and preparation study of the new chemical activator AHO of plant systemic acquired resistance, and the field experiment of five consecutive years was significant for the prevention and treatment of disease effects.

These honors have become a source of motivation for continual exploration and innovation in Professor Hao’s research team. Today, Professor Hao is still presiding over and undertaking many important scientific research projects such as the State Key Program of National Natural Science Foundation of China, research and development projects of new drugs and new pesticides. In spite of the heavy work, he devoted his energies to training students. He has trained 61 doctors, of whom 30 have been promoted to professors and become leaders in scientific research and academic institutions. More importantly, 36 of the former students serve in universities and research institutes among the Yunnan and Guizhou areas have made tremendous contributions to the development of the remote Southwest China.

Despite of the life-long outstanding achievements, Professor Hao continues to maintain a modest and low-key style. He often said that the achievements do not belong to himself, but the result of the team’s joint efforts. He can do more for the scientific research for the motherland and benefit the people is his highest wish.
